# Doppler Ultrasound Indices and Fetal Biometry as Prenatal Markers of SGA or Non-SGA Developmental Trajectories in Naturally Nutrient-Restricted Sheep Pregnancies from Patagonia

**DOI:** 10.3390/ani16101499

**Published:** 2026-05-14

**Authors:** Matías Araya, César Ulloa-Leal, Marcelo Ratto, Francisco Sales, Víctor H. Parraguez, Camila Sandoval

**Affiliations:** 1 Facultad de Ciencias Veterinarias y Pecuarias, Universidad de Chile, Santiago 8820808, Chile; matias.araya.b@ug.uchile.cl (M.A.); vparragu@uchile.cl (V.H.P.); 2 Facultad de Medicina Veterinaria, Universidad San Sebastián, Concepción 4081322, Chile; cesar.ulloa@uss.cl; 3 Facultad de Ciencias Veterinarias, Universidad Austral de Chile, Valdivia 5091000, Chile; marceloratto@uach.cl; 4 Instituto de Investigaciones Agropecuarias, Punta Arenas 6210489, Chile; fsales@inia.cl; 5 Facultad de Ciencias Agronómicas, Universidad de Chile, Santiago 8820808, Chile; 6 Facultad de Ciencias Veterinarias, Universidad de Concepción, Chillán 3800708, Chile

**Keywords:** maternal undernutrition, small for gestational age, Doppler, ultrasonography fetal biometry, resistance index, pulsatility index

## Abstract

Nutrient restriction (NR) increases small-for-gestational-age (SGA) offspring; however, some NR ewes deliver Non-SGA lambs. We evaluated whether fetal biometry and Doppler indices could distinguish divergent fetal growth trajectories. Ninety-five single-pregnant Corriedale ewes were assigned to NR grazing (*n =* 72) or supplemented Controls (*n* = 23) from gestational day (GD) 70 to 140. Fetal biparietal diameter (BPD), femur length (FL), thoracic height (TH), umbilical cord diameter (UCD), and resistance (RI) and pulsatility (PI) indices in umbilical (UA), cotyledonary (CA), and uterine (UtA) arteries were assessed at several GDs. Offspring within NR group was stratified by birth weight (BW) quartiles as SGA (*n* = 18) or Non-SGA (*n* = 18). At birth, BW differed (*p* < 0.05) among Control (4.95 ± 0.10 kg), Non-SGA (5.33 ± 0.06 kg), and SGA (3.79 ± 0.11 kg), with reduced BPD and FL in SGA lambs. Prenatal biometry did not differ. UA-RI at GD125 was higher in SGA (*p* < 0.005) and associated with BW (R^2^ = 0.15; *p* < 0.001). UtA indices were lower in SGA at GD110 and GD125 (*p* < 0.05) but weakly associated with BW (R^2^ ≤ 0.08). Doppler differences were detected before measurable growth divergence but have modest predictive value.

## Simple Summary

This study examined how poor nutrition during pregnancy affects lamb growth, since it has been stated that although undernourished sheep are more likely to have smaller-than-normal lambs (called small-for-gestational-age, or SGA), some still give birth to normal-sized offspring (Non-SGA), which may indicate a maternal adaptation to undernutrition. We studied whether prenatal ultrasound measurements of fetal growth and maternal-placental blood flow could provide information about the underlying causes leading to the production of SGA versus Non-SGA offspring in undernourished pregnancies, and if these parameters could be used as prenatal predictors of the SGA and Non-SGA outcomes. We found that differences in fetal size were not noticeable before birth, but changes in indicators of umbilical and uterine blood flow could be detected prenatally, and differed between lambs that were later born as SGA and those that were born as Non-SGA. Overall, ultrasound indicators of blood flow in uterine and umbilical vessels may provide early warning signs of fetal growth alterations. However, they are not reliable for accurately predicting which lambs will be born as SGA or Non-SGA.

## 1. Introduction

Pasture-based animal operations, and particularly rangeland systems, often face difficulties in meeting the nutritional requirements of animals because of insufficient forage quantity and quality. Worldwide, this scenario is likely to worsen as a consequence of climate change and its effects on agroclimatic conditions [1]. Variations in rainy and drought periods, temperature modifications, and increase in desertification are expected to have a negative impact on forage availability in several areas of the world [2,3]. These conditions negatively affect forage quality and quantity, leading to naturally occurring animal undernutrition. This situation becomes more critical during periods of increased nutritional requirements, such as gestation, when nutritional requirements of dams are augmented to support fetal and placental growth [4].

Gestational undernutrition leads to a reduction in fetal growth and birth weight, producing neonates that are small for gestational age (SGA) [5,6,7]. In humans, this classification refers to neonates that are below the 10th percentile, or below two standard deviations, for the expected birth weight or fetal length, at a given gestational age [8]. In animal models, different selection criteria have been used to define SGA animals, with all of them finally referring to offspring that are smaller than expected for the species at a specific gestational age or at birth [9]. Extensive research has been conducted in the field, and several studies have used sheep as a model of maternal NR and SGA offspring because of its value for both agricultural and biomedical research [10]. Insights from those models indicate that SGA offspring is at a higher risk of developing metabolic diseases in postnatal life [11,12,13,14]. From an animal production perspective, this condition may lead to increased mortality [15], altered growth performance, altered carcass composition, and potential detrimental effects in reproductive parameters such as puberty onset, expected fertility, and ovulation rate [16,17,18].

Most of the available studies using animal models of NR have considered fetuses from NR females as a single experimental group [7,14,19]. However, previous research in ovine models of NR has demonstrated the appearance of a broad range in the distribution of fetal weights within the NR groups. This has led to the identification of lambs presenting normal birth weight, regardless of being born to ewes that suffered the same gestational NR (Non-SGA offspring) [20,21,22]. The occurrence of SGA and Non-SGA offspring in NR pregnancies has been observed in different species [23], and also in a paradoxical model of overnourished adolescent sheep [24]. In previous observations of ovine flocks from rangeland systems in Patagonia, we have also identified the appearance of SGA and Non-SGA offspring born to ewes that were subject of a natural NR imposed by harsh environmental conditions.

Available data from the aforementioned studies suggest a differential metabolic programming between Non-SGA and SGA offspring which may determine differential postnatal health and performance [25]. Additionally, as neonatal survival is directly associated with birth weight [26], it could be anticipated that Non-SGA offspring will exhibit reduced mortality, regardless of being born to NR ewes. This is particularly relevant for ovine rangeland systems placed in harsh environments, such as southern Patagonia. In these areas, environmental temperatures are low during the lambing season, which may become even more challenging during cold waves, rain or snow storms [27]. As these are extensive systems, parturition occurs in grazing paddocks, usually without cover for the animals or possibilities to provide particular care to the neonates. As a consequence, neonatal mortality is high, which affects weaning percentage and the economical sustainability of these systems. From this perspective, the possibility of identifying the SGA and Non-SGA developmental trajectories prenatally could allow farmers to strategically prioritize their feeding resources for at-risk pregnancies. This would have a positive impact on NR ewes carrying SGA pregnancies, allowing them to fully cover their metabolic needs, supporting adequate placental development, as well as meeting the fetus nutrient demands, hence mitigating the SGA outcome and the associated consequences.

The literature derived from human SGA pregnancies indicates Doppler ultrasound exploration of placental and umbilical vessels as a reliable and non-invasive tool for the prenatal detection of this condition [28]. Specifically, it is reported that two indicators, the pulsatility (PI) and resistance (RI) indices, are increased in SGA pregnancies, particularly in the umbilical artery [29]. These Doppler indices are directly correlated with one another, and present an inverse correlation with tissue perfusion [30] and blood flow [31]. Thus, increased PI and RI in SGA pregnancies are ultimately indicating an altered perfusion and blood flow throughout the placenta, hence limiting nutrient and oxygen delivery to the fetuses, limiting intrauterine growth and development [32]. Once this limitation in fetal growth was manifested, it was prenatally assessed via B-mode ultrasound fetal biometry. Indicators, such as biparietal diameter, umbilical girth, femoral and tibial length, are correlated with lamb birth weight, presenting a value as eventual predictors of this condition [33].

The aforementioned techniques represent a potential non-invasive alternative for the prenatal identification of SGA pregnancies that could be applied under field conditions in rangeland ovine systems. However, while most of the available literature has demonstrated the utility of Doppler ultrasound assessment in humans, it remains unknown if the alterations leading to SGA offspring as a consequence of maternal NR will cause the same alterations in Doppler indicators in an ovine pregnancy. It is also unknown if Doppler indices and B-mode fetal biometry could be useful for a prenatal differentiation between Non-SGA and SGA developmental trajectories in pregnancies under natural NR. Hence, our objective was to evaluate whether fetal biometry and Doppler ultrasonography could be used to anticipate the appearance of Non-SGA and SGA fetuses in single-bearing ewes under natural NR conditions.

## 2. Materials and Methods

All animal procedures were evaluated and approved by the Institutional Animal Care and Use Committee of INIA (n° 01/2022). The studies were conducted in the sheep research unit of INIA Kampenaike, Magallanes Region, Chilean Patagonia (Lat 52°36′; Lon 70°56′).

### 2.1. Animal Management

A total of 120 Corriedale multiparous sheep were selected based on age (between 4 and 5 years) and average body condition score (BCS) of 2.5 points (ranging from two to three in a 1–5 scale). The reported BCS is below the standard recommendation of three points as an ideal condition. However, it is considered normal for ewes under the rangeland systems of Patagonia, where natural nutrient restriction occurs throughout the year. All females received two injections of prostaglandin F_2α_ (PGF_2α_) 11 days apart to synchronize estrus. Females detected in estrus were artificially inseminated using fresh semen from the same male in order to reduce genetic variability and avoid the male effect over birth weight. Briefly, semen was collected using an artificial vagina, evaluated for motility and concentration, and diluted in non-fat milk in a 1:4 proportion. Diluted semen was maintained in a warm bath during the insemination process. Ewes were cervically inseminated using a speculum to identify the cervix, and an insemination pipette was used to deposit the semen at the cervical opening. All inseminations were performed by the same trained operator. Ultrasound pregnancy diagnosis was performed at gestational day (GD) 70. Only singleton pregnancies (*n* = 95) were selected for this study. Animals grazed in natural pasture, in the same paddock from estrus synchronization to GD 140. At GD 140, ewes were placed in individual pens for lambing control in a 24/7 regime. Animals had ad libitum access to water throughout the experiment.

### 2.2. Treatments and Experimental Groups

Selected ewes were randomly assigned to either a nutrient-restricted (NR, *n* = 72, BCS 2.5 ± 0.5) or a control (Control, *n* = 23, BCS 2.5 ± 0.5) group. The NR group was maintained in a paddock under a continuous grazing regime with stocking rate of 0.9 ewes/ha, dry matter production of 520 kg/ha, CP content of 3.2%, and ME of 1.6 Mcal/Kg, representing the typical grazing conditions of rangeland systems in Chilean Patagonia. This feeding regime provided 54% CP and 73% ME of the National Research Council (NRC, 2007) requirements [34]. The Control group was maintained by grazing in the same paddock, but received a daily concentrate supplementation (Suralim^®^ Núcleo Punta Arenas, IANSA, Santiago, Chile, 22% CP, 2.85 Mcal/Kg ME) from GD 70 to GD 140, in an amount sufficient to provide 100% of CP requirements according to NRC (2007) recommendations [34]. A mobile pen was placed within the grazing paddock so that the Control group received the supplementation separately from the NR group without being removed from the paddock. The concentrate rations were adjusted according to maternal weights and gestational stage. From GD 140 to parturition, all animals were fed in their individual pens, receiving a daily ration composed of meadow hay (6.85% CP, 2.13 Mcal/Kg ME, 950 gr/day) and alfalfa hay (22.65% CP, 2.39 Mcal/Kg ME, 100 gr/day) which was provided divided into two administrations per day. Water was provided ad libitum for all animals. Maternal weight (MW) was recorded at GD 70, 90, 110, 125, and 140. Lamb birth weight (BW) was recorded at parturition. Animals born to NR ewes were separated into quartiles according to BW as previously described [20,21,25]. Briefly, lambs within the highest quartile were classified as the Non-SGA group (*n* = 18), while lambs within the lowest quartile formed the SGA group (*n* = 18). Typically, in human studies, SGA is defined as offspring below the 10th percentile of birth weight. However, the broad definition of SGA refers to offspring that is smaller or lighter than expected at a given gestational age [9]. From that basis, the aforementioned quartile approach allowed for a maximization of phenotypic contrast between our groups, while avoiding an exaggerated increase in the initial number of ewes. Offspring within the middle quartiles were not classified as either SGA or Non-SGA, hence they were not considered in statistical comparisons among experimental groups. All animals within the control group (*n* = 23) were considered for analysis. Quartile division was not applied to this group as previous observations indicated a minimal variation range in the BW of lambs born to control ewes. As quartile division was applied to lambs from NR ewes but not to lambs from Control ewes, the initial allocation of animals to the NR group was increased in order to reach a similar *n* number per experimental group (Control, Non-SGA and SGA).

### 2.3. Prenatal Evaluations—Fetal Biometry

Biparietal diameter (BPD), femoral length (FL), thorax height (TH), and umbilical cord diameter (UCD) were evaluated at GD 80, 95, 110, and 125 by B-mode ultrasound, using a SonoScape ProVet E2V2 (SonoScape, Medical Corp., Shenzhen, China) ultrasound device, and a 5 MHz transabdominal microconvex probe (SonoScape, Medical Corp., Shenzhen, China). Measurements were not performed at GD 140 due to technical limitations of the ultrasound probes that were available. Briefly, ewes were shorn in the abdominal area before scanning to improve contact between skin and the probe. All ultrasound examinations and imaging were performed by the same trained operator, in order to avoid personnel-related variations. Images were stored and subsequently measured using ImageJ 1.54r version (Wayne Rasband, National Institutes of Health, Bethesda, MD, USA). Each measurement was performed as described in previous studies. Briefly, BPD was measured by placing the calipers on the outer edge of one parietotemporal bone and the inner edge of the opposite [35]. FL was measured along the length of the ossified femoral diaphysis [36]. TH was measured as the distance from the sternum to the vertebral column at mid-heart level on a transverse thoracic section [37]. UCD was measured on a transverse section, away from its fetal insertion to standardize the measurement procedure. Measurements were obtained from all animals in the study, but further analyses considered only data from pregnancies leading to Control *(n* = 23), Non-SGA (*n* = 18) and SGA (*n* = 18) offspring, thus excluding NR animals that produced offspring within the middle quartiles for BW.

### 2.4. Prenatal Evaluations—Doppler Ultrasonography

Resistance (RI) and pulsatility (PI) indices were evaluated in umbilical (UA-RI and UA-PI), cotyledonary (CA-RI and CA-PI), and uterine (UtA-RI and UtA-PI) arteries at GD 80, 95, 110, 125, and 140 via spectral Doppler ultrasonography using the aforementioned equipment. The same trained operator performed all evaluations to avoid personnel-related variation. Assessment of UA was performed through the transabdominal examination from a longitudinal section of free loop of the umbilical cord using 5 MHz microconvex transducer and 12° insonation angle [38]. Doppler ultrasound indicators for CA and UtA were assessed using a 9 MHz transrectal transducer with 12° insonation angle [38], and ewes standing in the chute in every examination. Due to impossibility of marking the specific placentome that was evaluated at each GD, we could only standardize the assessment to CA by measuring Doppler parameters in the same location of the selected placentome each time. UtA assessment was performed ipsilateral to the fetus. Each Doppler examination provided curves with peak systolic velocity (PSV) and end diastolic velocity (EDV), from which the ultrasound unit automatically derived RI and PI. Measurements were obtained from all animals in the study, but further analyses considered only data from pregnancies leading to Control (*n* = 23), Non-SGA (*n* = 18) and SGA (*n* = 18) offspring, thus excluding NR animals that produced offspring within the middle quartiles for BW.

### 2.5. Postnatal Measurements

Immediately after lambing, offspring were weighed, individually identified, and neonatal BPD, FL, and TH were measured using a caliper. As mentioned previously, only lambs from NR group were divided into quartiles based on BW to form the Non-SGA and SGA groups. Therefore, measurements were obtained from all animals in the study, but further analyses considered only Control (*n* = 23), Non-SGA (*n* = 18) and SGA (*n* = 18) groups, thus excluding the lambs born to NR ewes which fell within the middle quartiles for BW.

### 2.6. Data Analyses

All results were analyzed using JMP^®^ version 19 (SAS Institute Inc., Cary, NC, USA). Sample size was determined by power analysis to find the minimum *n* number required to detect variations in BW. Data was evaluated for normality using Shapiro–Wilks test. Levene’s test was used to evaluate variance homogeneity. Comparison between groups for postnatal data was performed via one-way ANOVA followed by Tukey’s test as post hoc analysis. A generalized linear mixed-effects model was used for comparison between groups in data collected multiple times during gestation. The effect of group, time and their interaction was considered in the model. Fetal sex was not included in the model as its effect was not significant. Statistical significance was defined for *p*-value ≤ 0.05, while 0.05 < *p*-value < 0.1 was considered statistical tendency. Results are presented as mean ± standard error of the mean (SEM). All the mentioned analysis compared SGA, Non-SGA and Control groups.

A linear regression analysis was performed for the prenatal variables that showed significant differences between groups in the aforementioned analysis. Particularly for this analysis, regressions were conducted using the entire dataset instead of the extreme quartiles for each variable against birth weight. Determination coefficients (R^2^) were used to assess the percentage of variability in birth weight that was explained by each of the selected variables, as an initial assessment of their robustness as prenatal predictors of SGA and Non-SGA offspring.

## 3. Results

### 3.1. Lamb Birth Weight

**Figure 1:**
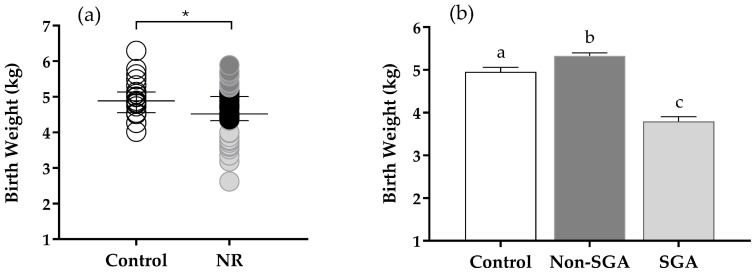
(**a**) Birth weight (BW) for Control (*n* = 23) and nutrient-restricted (NR) (*n* = 72) lambs. Animals within the NR group were divided into quartiles based on BW. Highest quartile (dark gray circles) formed the Non-SGA group (*n* = 18). Lowest quartile (light gray circles) formed the SGA group (*n* = 18) * indicates statistical significance. (**b**) Mean BW for Control, Non-SGA, and SGA group. Bars with different letters are significantly different (*p* < 0.05).

The mean BW was higher in lambs born to Control (4.95 ± 0.1) compared to those born to NR (4.56 ± 0.08) ewes (*p* < 0.05) (Figure 1a). Once quartile separation was applied on NR lambs, all groups differed from each other (*p* < 0.05). The Non-SGA group was the heaviest (5.33 ± 0.06), followed by Control (4.95 ± 0.1), and SGA (3.79 ± 0.11) (Figure 1b).

### 3.2. Maternal Weight Across Pregnancy

**Figure 2:**
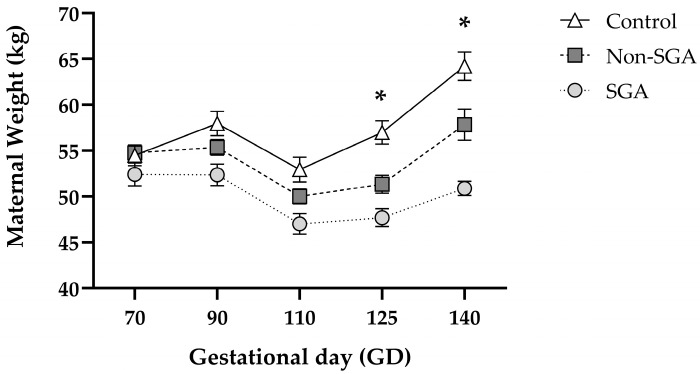
Maternal weight (MW) through gestation in Control (*n* = 23), Non-SGA (*n* = 18), and SGA (*n* = 18) groups. * indicates significant differences between SGA and Control at GD125, and among all groups at GD 140 (*p* < 0.05).

Maternal weight did not differ between groups at GD 70, 90, and 110. However, at GD 125, the SGA group weighed less than Control (47.68 ± 1.37 vs. 56.9 ± 1.22, respectively) (*p* < 0.05), while Non-SGA (51.34 ± 1.41) did not differ from either group (*p* > 0.05). All groups differed at GD 140, with mean weights of 64.2 ± 1.55, 57.8 ± 1.63, and 50.88 ± 1.92 for Control, Non-SGA, and SGA, respectively (*p* < 0.05) (Figure 2).

### 3.3. Fetal and Newborn Biometry

**Figure 3:**
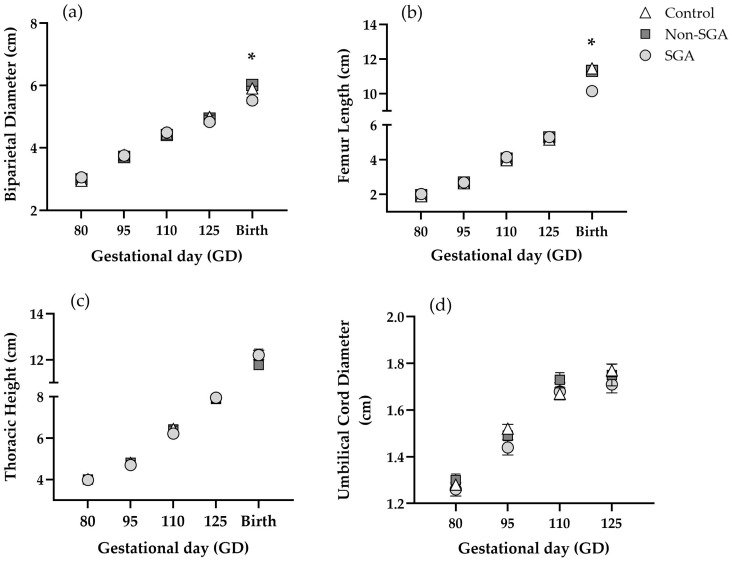
Fetal and postpartum biometry of (**a**) biparietal diameter (BPD), (**b**) femur length (FL) and (**c**) thorax height (TH), and (**d**) umbilical cord diameter (UCD) for Control (*n* = 23), Non-SGA (*n* = 18), and SGA (*n* = 18) groups. Gestational measurements were obtained by B-mode ultrasound, whereas postpartum data were collected using a manual caliper. UCD was not measured at birth. * indicates significant differences (*p* < 0.05).

All fetal ultrasound measurements showed a consistent increase as pregnancy progressed (*p* < 0.0001) (Figure 3). There were no differences between groups for either BPD (Figure 3a, Table S1), FL (Figure 3b, Table S2), TH (Figure 3c, Table S3), or UCD (Figure 3d, Table S4) at any gestational age (*p* > 0.05).

Postpartum BPD was significantly smaller for the SGA group (5.52 ± 0.09) compared to both Control (5.9 ± 0.07) and Non-SGA groups (6.02 ± 0.08) (*p* < 0.001) (Figure 3a—birth). The same pattern was observed for FL, with the SGA group (10.14 ± 0.09) being significantly smaller than both Control (11.46 ± 0.17) and Non-SGA (11.32 ± 0.11) groups (*p* < 0.001) (Figure 3b—birth). Postpartum TH did not differ among groups (*p* > 0.05) (Figure 3c—birth).

### 3.4. Doppler Color Ultrasonography

#### 3.4.1. Umbilical Artery Doppler

**Figure 4:**
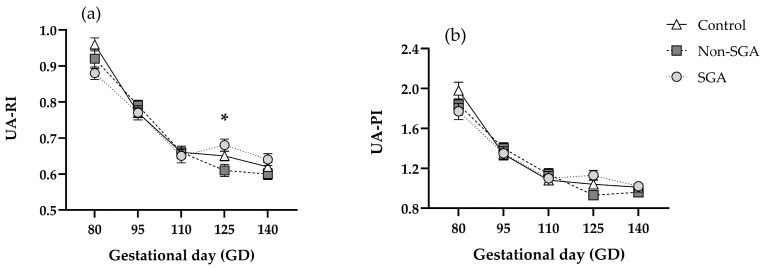
Umbilical artery resistance index (UA-RI) (**a**) and pulsatility index (UA-PI) (**b**) in Control (*n* = 23), Non-SGA (*n* = 18), and SGA (*n* = 18) groups throughout pregnancy. * indicates significant differences (*p* < 0.05).

Both RI and PI were significantly reduced over time, in accordance with gestational progression (Figure 4). Results for RI at GD 125 showed significant differences between SGA (0.68 ± 0.01) and Non-SGA (0.61 ± 0.01) groups (*p* < 0.01), and a tendency for differences between Control (0.62 ± 0.01) and Non-SGA groups (*p* < 0.1) (Figure 4a, Table S5). No differences were found for PI (*p* > 0.05) at any gestational age (Figure 4b, Table S6). 

#### 3.4.2. Cotyledonary Artery Doppler

**Figure 5:**
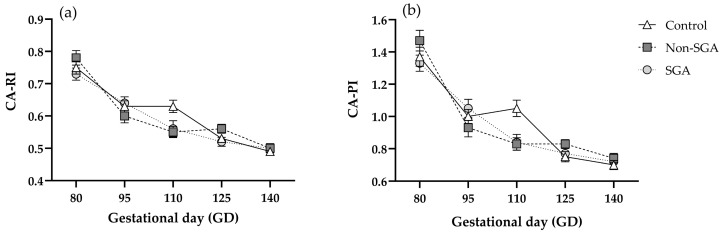
Cotyledonary artery resistance index (CA-RI) (**a**) and pulsatility index (CA-PI) (**b**) in Control (*n* = 23), Non-SGA (*n* = 18), and SGA (*n* = 18) groups. No differences were observed at any gestational age (*p* > 0.05).

Both RI and PI were significantly reduced throughout gestation (Figure 5, Tables S7 and S8). No differences were found among groups at any gestational age (*p* > 0.05).

#### 3.4.3. Uterine Artery Doppler

**Figure 6:**
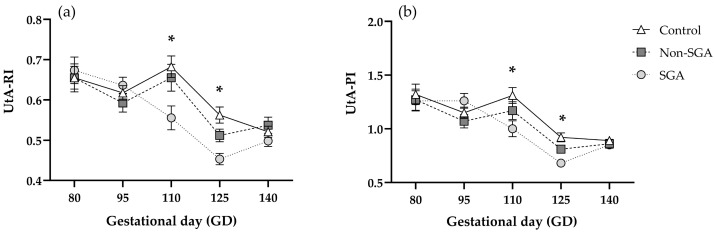
Uterine artery resistance index (UtA-RI) (**a**) and pulsatility index (UtA-PI) (**b**) for Control (*n* = 23), Non-SGA (*n* = 18), and SGA (*n* = 18) groups during gestation. * indicates significant differences (*p* < 0.05).

Both UtA-RI and UtA-PI were significantly reduced throughout gestation (*p* < 0.0001) (Figure 6). Measurements for RI at GD 110 were lower for the SGA group (0.55 ± 0.02) compared to both Non-SGA (0.65 ± 0.03) and Control (0.68 ± 0.02) (*p* < 0.001) (Figure 6a, Table S9). At GD 125, the SGA group (0.45 ± 0.01) differed significantly from Control (0.57 ± 0.02), while Non-SGA (0.52 ± 0.01) was intermediate between groups (*p* < 0.05) (Figure 6a).

**Figure 7:**
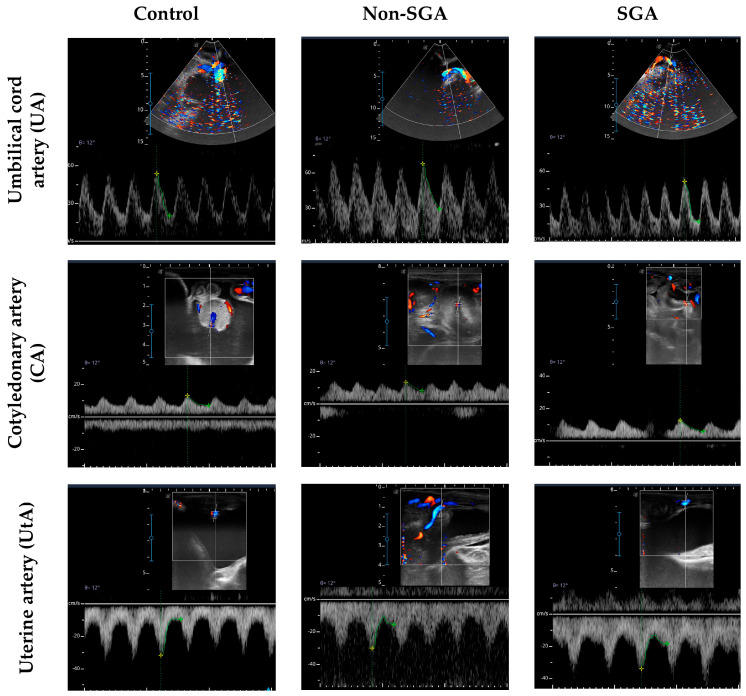
Representative images of Doppler evaluation on umbilical cord artery (UA), cotyledonary artery (CA) and uterine artery (UtA) for the Control, Non-SGA, and SGA groups at gestational day (GD).

Similar results were found for PI at GD 110, where values were lower in SGA (1.00 ± 0.07) than the Control (1.31 ± 0.07) group, while Non-SGA (1.17 ± 0.08) was intermediate (*p* < 0.05). Measurements of PI at GD 125 showed lower values for the SGA group (0.69 ± 0.02) than Control (0.98 ± 0.04) (*p* < 0.001), while Non-SGA (0.84 ± 0.03) was intermediate between groups (Figure 6b, Table S10). Representative images of Doppler evaluations are presented in Figure 7.

### 3.5. Linear Regression Between Prenatal Significant Variables and Birth Weight

**Figure 8:**
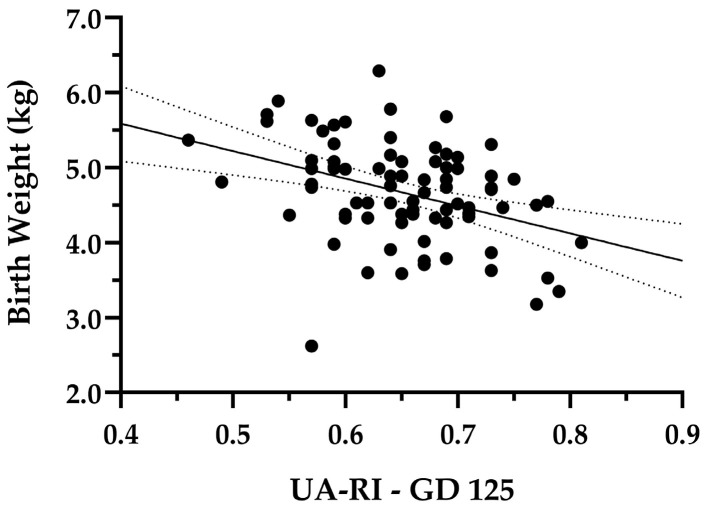
Linear regression between lamb birth weight (BW) and umbilical artery resistance index (UA-RI) at GD 125 (R^2^ = 0.15, *p* < 0.001).

**Figure 9:**
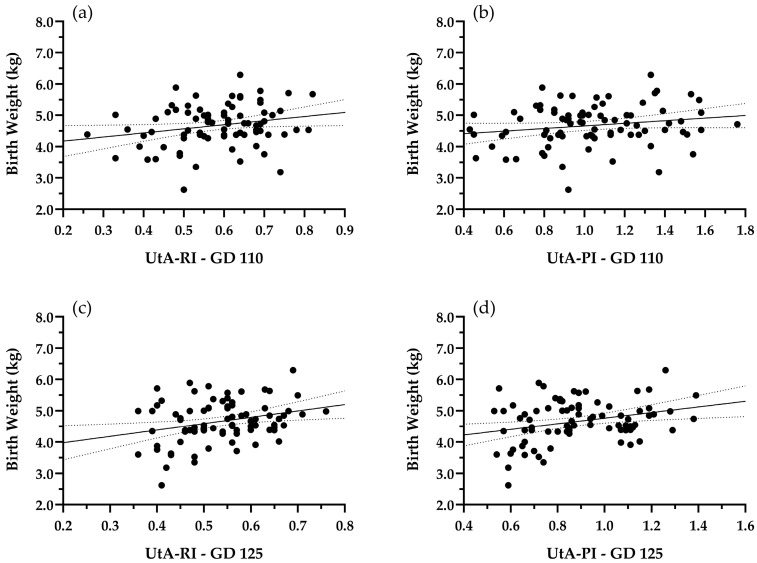
Linear regressions between (**a**) lamb birth weight (BW) and uterine artery resistance index at GD 110 (UtA-RI—GD 110) (R^2^ = 0.05, *p* < 0.05); (**b**) lamb birth weight (BW) and uterine artery pulsatility index at GD 110 (UtA-PI—110) (R^2^ = 0.03, *p* > 0.05); (**c**) lamb birth weight (BW) and uterine artery resistance index at GD 125 (UtA-RI—GD 125) (R^2^ = 0.07, *p* < 0.05); and (**d**) lamb birth weight (BW) and uterine artery pulsatility at GD 125 (UtA-PI—GD 125) (R^2^ = 0.08, *p* < 0.05).

We selected the entire dataset of UA-RI at GD 125, and both UtA-RI and UtA-PI at GD 110 and 125 to perform a correlation analysis, as those variables presented significant differences between groups according to the aforementioned analysis. A linear regression model was run between each of the mentioned variables and BW. The model for UA-RI GD125 and lamb BW was significant (*p* < 0.001), with a determination coefficient (R^2^) of 0.15 (Figure 8). At GD 110, results for UtA-RI were significant with *p* < 0.05, and R^2^ of 0.05 (Figure 9a), and results for UtA-PI presented an R^2^ of 0.03 (Figure 9b), with linear regression that was not significant (*p* > 0.05). Finally, results for UtA-RI and UtA-PI at GD125 were significant, with *p* < 0.05 and R^2^ of 0.07 and 0.08, respectively (Figure 9c,d).

## 4. Discussion

The present study aimed to evaluate whether fetal B-mode biometry and Doppler color ultrasonography could be used to prenatally differentiate Non-SGA and SGA gestations, under conditions of natural NR. Major findings indicate that while SGA and Non-SGA developmental trajectories were found, they were not prenatally detected via ultrasound biometry, but were associated with differential responses in placental vascular resistance.

The appearance of SGA and Non-SGA developmental trajectories in response to artificially imposed maternal NR has been previously reported in sheep [21,22,25,39]. Our investigation studied the same phenomenon, but under natural NR imposed by environmental hardship, and comparing offspring falling within extreme quartiles (SGA vs. Non-SGA), in order to maximize phenotypic contrast within lambs born to NR ewes. We suggest that using animals from a region where sheep flocks have been exposed to nutritional hardship for several generations may lead to the appearance of particular maternal adaptations leading to the divergent SAG and Non-SGA phenotypes. This may explain the fact that NR pregnancies under a Non-SGA developmental trajectory led to a higher BW than observed in the SGA and Control groups. The latter is reinforced by the fact that MW shows divergent trajectories at GD 125, where ewes leading to SGA offspring were lighter than Control animals, and at GD 140, where all groups differed with mothers of SGA lambs being lighter than both mothers of Control and Non-SGA offspring. This likely indicates a differential maternal adaptation allowing the ewes producing Non-SGA lambs to better cope with nutritional hardship. This may be associated with a maternal lipid metabolism adaptation, as previous research has found increased plasma non-esterified fatty acids (NEFAs) in NR ewes leading to Non-SGA versus SGA offspring [40]. The particular metabolic differences that underpin these maternal adaptations in our model are of high interest and are currently being investigated, but are beyond the scope of the present work.

Previous studies have provided relevant insights into differential placental adaptations allowing for enhanced nutrient delivery towards the fetus in Non-SGA versus SGA pregnancies. For example, Non-SGA pregnancies exhibited preserved placentome size and exchange surface, and a selective upregulation of amino acid transporters [20]. Reduced immune activation and selective upregulation of membrane receptor signaling in Non-SGA placentas has also been reported [22]. However, knowledge about non-invasive prenatal markers for an early detection of SGA and Non-SGA pregnancies is still lacking. We evaluated fetal ultrasound biometry as a non-invasive tool that may be suitable for these purposes. In agreement with previous reports [41], all biometric measurements increased throughout gestation. However, no differences were found at either GD 80, 95, 110, or 125 for BPD, FL, TH, and UCD. By contrast, results from a model of fetal growth restriction, induced by overfeeding adolescent ewes, indicated reduced fetal abdominal circumference and femoral length at GD 98, reduced renal volume and tibial length at GD 105, and smaller biparietal diameter from GD 112 in advance [33]. This study also found a variety of fetal weights within the overnourished group and separated them into SGA and Non-SGA offspring. Femoral length, tibial length, renal volume, and biparietal diameter were smaller in SGA versus Non-SGA groups at GD 126 [33]. Another model of intrauterine growth restriction, induced by surgical ligation of umbilical artery in sheep, also reported a reduction in biometrical measurements (crown-rump length, abdominal circumference, femur length, and tibial length) at GD 127 [42]. These results differ from our data, likely due to the different models used to induce the impairment of fetal development. The severity of placental insufficiency imposed by surgical intervention or the overfeeding model may impact fetal growth earlier than our current model of NR. Regardless, the most accelerated fetal growth occurs in the last third of gestation [43] and previous authors have stated that by GD 121, the ovine fetus reaches about 70% of total growth [33], revealing the relevance of the last 26 days of gestation in fetal growth. Given that we observed reduced newborn weight, biparietal diameter, and femoral length in the SGA group, we conclude that the physical differences in our model manifested at some point between GD 125 and birth, which is consistent with the period of most accelerated fetal growth.

Results from placental Doppler exploration indicate that RI and PI in UA, CA, and UtA showed a progressive decrease throughout gestation, regardless of the experimental group. This agrees with the previous literature from different mammalian species, which indicates that the reduction in RI and PI indices during pregnancy reflects an essential physiological adaptation to enhance blood flow towards fetal–placental tissues [30,44,45,46,47,48]. Regarding the prenatal differentiation of SGA and Non-SGA developmental trajectories, we found that RI and PI did not differ between groups for CA. We acknowledge that a potential limitation of the CA assessment was the inability to consistently evaluate the same placentome across time points, which may have contributed to increased variability in the dataset. To our knowledge, there are no non-invasive methods currently available to enable consistent evaluation of the same placentome over time, which we recognize as a limitation and a key area for future methodological improvement.

Results for UA indicate an increase in RI in the SGA group compared to Non-SGA and Control at GD 125. The latter has been previously reported in the literature in other species. For example, in humans, increases in RI and PI in umbilical artery are recognized as prenatal indicators of intrauterine growth restriction [28]. Studies performed in pregnant cattle under a 60% NR during the first 140 GD showed an increase in RI in the umbilical artery of fetuses within the restricted group compared to control-fed animals [44]. Similarly, previous reports from SGA developmental trajectories in a model of overnourished adolescent ewe have also associated increases in RI and PI indices with impaired intrauterine growth [33]. Interestingly, these authors also demonstrated that alterations in umbilical artery Doppler indices were evident before the onset of significant growth restriction. This implies that placental hemodynamic alterations can be considered among the subjacent causes leading to SGA offspring, and as an early indicator of an impaired developmental trajectory, suggesting that differential placental vascular responses preceded the actual divergence in fetal growth within the NR group.

Interestingly, previous research in near-term fetal sheep demonstrated that UA diastolic and mean velocities, PI, and RI, closely track directly measured placental blood flow and resistance [29], demonstrating that increased PI and RI correlates with reduced placental blood flow. In the context of our model, this could indicate that maternal NR leads to placental insufficiency in SGA pregnancies, impairing placental blood flow and nutrient delivery to the fetuses, thus explaining the impaired fetal growth. In fact, previous studies by our group in Magellan Patagonia described that, in sheep, maternal undernutrition generates placental insufficiency, fetal hypoxemia, and oxidative stress, leading to low birth weight, both in singleton and twin pregnancies [49,50]. Furthermore, the study of Acharya et al. [29] also demonstrated that UA Doppler primarily reflects downstream placental vascular load. Hence, it is most likely that a major differential adaptive response leading to SGA or Non-SGA developmental trajectories lies on a particular placental vascular adaptation that mitigates placental insufficiency in the Non-SGA group.

To our knowledge, this is the first report indicating that differential developmental trajectories can be prenatally differentiated throughout the umbilical Doppler in a model of natural maternal NR in sheep. The underlying causes leading to this differential response remain to be investigated, but the previous literature indicates that the fetal–placental responses to blood flow are locally regulated as those vessels are not under the regulation of autonomous nervous system [51,52]. Key factors that act as local vasodilators are the mechanical effect of blood flow, fluid shear stress, and endothelial vasoactive mediators [52]. Nitric oxide is a potent vasodilator produced by the enzyme endothelial nitric oxide synthase (eNOS), and it is a major local vasodilator in fetal–placental tissues [53,54]. The major source for nitric oxide synthesis in endothelial cells is amino acid arginine [54]. Previous research performed in a model of maternal NR in sheep, where SGA and Non-SGA fetuses were also identified, showed a reduction in the total content of arginine in the amniotic fluid of SGA placentas compared to Non-SGA [20]. It is feasible that this also occurs in the current model. This could be an underlying cause for an impaired endothelial capacity to synthetize NO due to a scarcity of its precursor in the SGA group. This would affect local vascular relaxation, hence increasing resistance to blood flow and, accordingly, rising the RI index as we have seen in this study. The opposite would occur within the Non-SGA group, which may present an enhanced placental blood flow that would allow for improved delivery of nutrients towards the fetus, despite nutrient restriction. These specific adaptations are of particular interest and remain to be investigated in our model.

Conversely, results for UtA-RI and UtA-PI showed an opposite response as the SGA group presented a lower RI and PI at GD 110 and 125, compared to the Non-SGA and Control groups. To our knowledge, the previous literature exploring hemodynamic responses of UtA via Doppler ultrasound is currently lacking in ovine models of maternal NR. However, insights from human studies indicate that elevated UtA-RI and UtA-PI are associated with fetal growth restriction, even recognizing this parameter as a predictive tool for the generation of SGA offspring [55,56]. Increases in RI or PI in the uterine artery would lead to higher resistance to blood flow towards the uterus and placenta, finally limiting the perfusion of fetal–placental tissues, and impairing fetal growth [57]. Nevertheless, these studies are mostly associated with pathological conditions such as pre-eclampsia, which lead to SGA offspring due to mechanisms that may not be comparable to those exerted by NR. Pre-eclampsia is among the major causes of SGA offspring in humans, which is characterized by generalized maternal hypertension. The latter is most likely induced by a failure in the remodeling of spiral arteries, which are essential for the correct human placentation [56]. Hence, finding increased UtA-RI and PI in human SGA pregnancies is expected based on the pathological underlying causes. However, our model represents differential maternal responses to natural and long-term NR, leading to SGA and Non-SGA offspring, rather than responses to pathological placental insufficiency. Another study performed in cattle evaluated the impact of a 70% maternal NR during late gestation on UtA Doppler indices and birth weight. No effect of maternal NR was found on either UtA-RI or UtA-PI, and birth weight was also unaffected [58]. To our knowledge, the aforementioned study is the closest to our current work; however, major differences regarding the species, the severity of NR, and the pregnancy stage in which it was applied could explain why those results are not concordant with our findings.

We suggest that the decreased UtA-RI and UtA-PI observed within the SGA group are indicative of a maternal compensation attempt to face nutritional hardship by adjusting vascular resistance of this vessel. This mechanism would increase blood flow towards utero-placental structures, in an effort to protect fetal perfusion, nutrient delivery, and growth. However, according to our data, this maternal compensatory mechanism does not succeed since UA responds in the opposite manner.

Furthermore, we ran a linear regression analysis to evaluate if the Doppler indices that presented significant differences between groups could be used as prenatal predictors of birth weight. Overall, the regressions between UtA-RI and PI, at both GD110 and 125, presented an extremely low R^2^, indicating that these variables alone explain very little of the BW variation that we observed before applying the quartile division of the data. Results for UA-RI at GD125 presented R^2^ = 0.15, indicating that this variable alone explains about 15% of the BW variation that we observed. The relatively low R^2^ value suggests that, while UA-RI at GD 125 is associated with the underlying causes leading to either SGA or Non-SGA developmental trajectories, its explanatory power as a prenatal predictor of this response is modest. This limits its use as a standalone early detector of SGA versus Non-SGA pregnancies at the individual level and at the flock-level. Further integration with additional biomarkers is warranted in order to achieve the goal of prenatally identifying compromised pregnancies, providing a tool to make flock-level decisions and establish mitigation strategies.

## 5. Conclusions

This work identified the appearance of SGA and Non-SGA developmental paths in singleton pregnancies from ewes under natural NR. These divergent trajectories were not prenatally identified by routine B-mode ultrasound fetal biometry. However, Doppler color assessment showed reduced UtA-RI and UtA-PI at GD 110 and 125, and increased UA-RI at GD 125 in SGA offspring. This likely indicates a maternal compensation attempt within the SGA group, which is not mirrored within the fetoplacental circulation as our results indicate an impaired blood blow at the umbilical level in SGA fetuses. Regardless of the identified differential Doppler hallmarks, UtA-RI and UtA-PI explained very little of BW variations, while UA-RI presented a modest power, explaining 15% of the variability in BW.

## Data Availability

Data is available by request to the corresponding author of the manuscript.

## Supplementary Materials

The following supporting information can be downloaded at https://www.mdpi.com/article/10.3390/ani16101499/s1, Table S1. Biparietal diameter (BPD) in Control, Non-SGA and SGA groups at gestational days 80, 95, 110, 125 and at birth. Table S2. Femur Length (FL) in Control, Non-SGA and SGA groups at gestational days 80, 95, 110, 125 and at birth. Table S3. Thoracic height (TH) in Control, Non-SGA and SGA groups at gestational days 80, 95, 110, 125 and at birth. Table S4. Umbilical cord diameter (UCD) in Control, Non-SGA and SGA groups at gestational days 80, 95, 110 and 125. Table S5. RI-UA in Control, Non-SGA and SGA groups at gestational days 80, 95, 110, 125 and 140. Table S6. PI-UA in Control, Non-SGA and SGA groups at gestational days 80, 95, 110, 125 and 140. Table S7. RI-CA in Control, Non-SGA and SGA groups at gestational days 80, 95, 110, 125 and 140. Table S8. PI-CA in Control, Non-SGA and SGA groups at gestational days 80, 95, 110, 125 and 140. Table S9. RI-UtA in Control, Non-SGA and SGA groups at gestational days 80, 95, 110, 125 and 140. Table S10. PI-UtA in Control, Non-SGA and SGA groups at gestational days 80, 95, 110, 125 and 140.

## Abbreviations

The following abbreviations are used in this manuscript:

NR	Nutrient Restriction
SGA	Small for Gestational Age
Non-SGA	Non-Small for Gestational Age
GD	Gestational Day
BW	Birth Weight
RI	Resistance Index
PI	Pulsatility Index
UA	Umbilical Artery
CA	Cotyledonary Artery
UtA	Uterine Artery
BPD	Biparietal Diameter
FL	Femoral Length
TH	Thoracic Height
UCD	Umbilical Cord Diameter
