# Supplementary material for: Doppler Ultrasound Indices and Fetal Biometry as Prenatal Markers of SGA or Non-SGA Developmental Trajectories in Naturally Nutrient-Restricted Sheep Pregnancies from Patagonia
Source: Animals (Basel). 2026 May 14;16(10):1499. doi: 10.3390/ani16101499 (PMC13203453; doi:10.3390/ani16101499)
Supplement: Supplementary file 1 [file animals-16-01499-s001.zip › animals-4241760-supplementary.pdf]

-Table S1. Biparietal diameter (BPD) in Control, Non-SGA and SGA groups at gestational days 80, 95, 110, 125 and at birth.

Gestational Day	Control	Non-SGA	SGA
80	2.95±0.04 [a]	3.00±0.03 [a]	3.06±0.05 [a]
95	3.77±0.02 [a]	3.71±0.04 [a]	3.76±0.03 [a]
110	4.46±0.06 [a]	4.41±0.04 [a]	4.49±0.05 [a]
125	4.99±0.06 [a]	4.94±0.05 [a]	4.83±0.06 [a]
Birth	5.91±0.07 [a]	6.02±0.08 [a]	5.52±0.09 [b]

Different superscripts indicate significant differences ($p<0.05$)

-Table S2. Femur Length (FL) in Control, Non-SGA and SGA groups at gestational days 80, 95, 110, 125 and at birth.

Gestational Day	Control	Non-SGA	SGA
80	1.90±0.05 [a]	1.96±0.04 [a]	2.02±0.07 [a]
95	2.64±0.02 [a]	2.69±0.06 [a]	2.69±0.06 [a]
110	3.98±0.07 [a]	4.06±0.05 [a]	4.15±0.08 [a]
125	5.16±0.10 [a]	5.28±0.11 [a]	5.30±0.09 [a]
Birth	11.47±0.17 [a]	11.32±0.11 [a]	10.14±0.09 [b]

Different superscripts indicate significant differences ($p<0.05$)

-Table S3. Thoracic height (TH) in Control, Non-SGA and SGA groups at gestational days 80, 95, 110, 125 and at birth.

Gestational Day	Control	Non-SGA	SGA
80	3.98±0.05 [a]	4.06±0.08 [a]	4.00±0.12 [a]
95	4.70±0.04 [a]	4.85±0.06 [a]	4.81±0.06 [a]
110	6.22±0.07 [a]	6.49±0.08 [a]	6.42±0.06 [a]
125	7.95±0.06 [a]	7.93±0.07 [a]	7.89±0.07 [a]
Birth	12.20±0.20 [a]	12.21±0.24 [a]	11.77±0.16 [a]

Different superscripts indicate significant differences ($p<0.05$)

-Table S4. Umbilical cord diameter (UCD) in Control, Non-SGA and SGA groups at gestational days 80, 95, 110 and 125.

Gestational Day	Control	Non-SGA	SGA
80	1.28±0.02 [a]	1.30±0.02 [a]	1.26±0.02 [a]
95	1.52±0.01 [a]	1.49±0.02 [a]	1.44±0.03 [a]
110	1.67±0.02 [a]	1.73±0.03 [a]	1.68±0.03 [a]
125	1.77±0.02 [a]	1.75±0.04 [a]	1.71±0.03 [a]

Different superscripts indicate significant differences ($p<0.05$)

-Table S5. RI-UA in Control, Non-SGA and SGA groups at gestational days 80, 95, 110, 125 and 140.

Gestational Day	Control	Non-SGA	SGA
80	0.96±0.01[a]	0.92±0.02[a]	0.88±0.01[a]
95	0.77±0.01[a]	0.79±0.01[a]	0.77±0.01[a]
110	0.66±0.01[a]	0.66±0.01[a]	0.65±0.01[a]
125	0.65±0.01[ab]	0.61±0.01[a]	0.68±0.01[b]
140	0.62±0.01[a]	0.60±0.01[a]	0.64±0.01[a]

Different superscripts indicate significant differences ($p<0.05$)

-Table S6. PI-UA in Control, Non-SGA and SGA groups at gestational days 80, 95, 110, 125 and 140.

Gestational Day	Control	Non-SGA	SGA
80	1.98±0.08[a]	1.84±0.09[a]	1.77±0.08[a]
95	1.34±0.05[a]	1.40±0.05[a]	1.35±0.06[a]
110	1.08±0.03[a]	1.14±0.05[a]	1.10±0.06[a]
125	1.04±0.04[a]	0.93±0.04[a]	1.13±0.04[a]
140	1.01±0.03[a]	0.96±0.03[a]	1.01±0.03[a]

Different superscripts indicate significant differences ($p<0.05$)

-Table S7. RI-CA in Control, Non-SGA and SGA groups at gestational days 80, 95, 110, 125 and 140.

Gestational Day	Control	Non-SGA	SGA
80	0.75±0.02[a]	0.78±0.02[a]	0.73±0.01[a]
95	0.63±0.01[a]	0.60±0.02[a]	0.64±0.01[a]
110	0.63±0.01[a]	0.55±0.01[a]	0.56±0.02[a]
125	0.53±0.01[a]	0.56±0.01[a]	0.52±0.01[a]
140	0.49±0.01[a]	0.50±0.01[a]	0.50±0.01[a]

Different superscripts indicate significant differences ($p<0.05$)

-Table S8. PI-CA in Control, Non-SGA and SGA groups at gestational days 80, 95, 110, 125 and 140.

Gestational Day	Control	Non-SGA	SGA
80	1.37±0.05 [a]	1.47±0.06 [a]	1.33±0.05 [a]
95	1.00±0.04 [a]	0.93±0.05 [a]	1.05±0.05 [a]
110	1.05±0.05 [a]	0.83±0.03 [a]	0.84±0.04 [a]
125	0.75±0.03 [a]	0.83±0.02 [a]	0.77±0.03 [a]
140	0.70±0.02 [a]	0.74±0.03 [a]	0.72±0.02 [a]

Different superscripts indicate significant differences ($p<0.05$)

-Table S9. RI-UtA in Control, Non-SGA and SGA groups at gestational days 80, 95, 110, 125 and 140.

Gestational Day	Control	Non-SGA	SGA
80	0.65±0.02 [a]	0.65±0.03 [a]	0.67±0.03 [a]
95	0.61±0.01 [a]	0.59±0.02 [a]	0.63±0.02 [a]
110	0.68 ±0.02[a]	0.65±0.03[a]	0.55±0.02[b]
125	0.57 ±0.02[a]	0.52±0.01[ab]	0.45±0.01[b]
140	0.52±0.01 [a]	0.53±0.01[a]	0.50±0.01[a]

Different superscripts indicate significant differences ($p<0.05$)

-Table S10. PI-UtA in Control, Non-SGA and SGA groups at gestational days 80, 95, 110, 125 and 140.

Gestational Day	Control	Non-SGA	SGA
80	1.32±0.09 [a]	1.27±0.09 [a]	1.26±0.08 [a]
95	1.15±0.05 [a]	1.07±0.06 [a]	1.26±0.06 [a]
110	1.31±0.07 [a]	1.17±0.08 [ab]	1.00±0.07 [b]
125	0.98±0.04[a]	0.84±0.03[ab]	0.69±0.02[b]
140	0.89±0.03 [a]	0.86±0.03 [a]	0.85±0.03 [a]

Different superscripts indicate significant differences ($p<0.05$)
